# NAD^+^ homeostasis in human health and disease

**DOI:** 10.15252/emmm.202113943

**Published:** 2021-05-27

**Authors:** Rubén Zapata‐Pérez, Ronald J A Wanders, Clara D M van Karnebeek, Riekelt H Houtkooper

**Affiliations:** ^1^ Laboratory Genetic Metabolic Diseases Amsterdam Gastroenterology, Endocrinology, and Metabolism (AGEM) Amsterdam Cardiovascular Sciences (ACS) Amsterdam UMC University of Amsterdam Amsterdam The Netherlands; ^2^ Department of Pediatrics Amsterdam UMC University of Amsterdam Amsterdam The Netherlands; ^3^ Department of Pediatrics (Metabolic Diseases) Radboud Centre for Mitochondrial Medicine Amalia Children’s Hospital Radboud University Medical Center Nijmegen The Netherlands; ^4^ On behalf of ‘United for Metabolic Diseases’ Amsterdam The Netherlands

**Keywords:** disease, metabolism, NAD^+^, NAD^+^ homeostasis, therapy, Metabolism

## Abstract

Depletion of nicotinamide adenine dinucleotide (NAD^+^), a central redox cofactor and the substrate of key metabolic enzymes, is the causative factor of a number of inherited and acquired diseases in humans. Primary deficiencies of NAD^+^ homeostasis are the result of impaired biosynthesis, while secondary deficiencies can arise due to other factors affecting NAD^+^ homeostasis, such as increased NAD^+^ consumption or dietary deficiency of its vitamin B3 precursors. NAD^+^ depletion can manifest in a wide variety of pathological phenotypes, ranging from rare inherited defects, characterized by congenital malformations, retinal degeneration, and/or encephalopathy, to more common multifactorial, often age‐related, diseases. Here, we discuss NAD^+^ biochemistry and metabolism and provide an overview of the etiology and pathological consequences of alterations of the NAD^+^ metabolism in humans. Finally, we discuss the state of the art of the potential therapeutic implications of NAD^+^ repletion for boosting health as well as treating rare and common diseases, and the possibilities to achieve this by means of the different NAD^+^‐enhancing agents.

## Graphical abstract

This comprehensive review discusses pathological consequences of NAD^+^ metabolism alterations and the therapeutic potential of NAD^+^ enhancers.

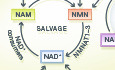

**Glossary**
Genetic disorder: A disease caused by changes (mutations) in the DNA sequence of an individual. These mutations can occur in one or in multiple genes.
Leber Congenital Amaurosis (LCA): Rare inherited eye disorder primarily affecting the retina. LCA symptoms include severe visual impairment, involuntary movement of the eyes (nystagmus), and/or increased sensitivity to light (photophobia).
Mitochondria: Subcellular compartments (organelles) found in the cytoplasm of eukaryotic cells equipped with the enzymatic machinery to degrade fatty acids, sugar‐derived substrates, and amino acids to CO_2_ and H_2_O to synthesize ATP through aerobic respiration via a process called oxidative phosphorylation.
NAD^+^ precursor: Molecule that can be converted into NAD^+^ through a series of reactions inside cells. To date, established NAD^+^ precursors are tryptophan, nicotinic acid (NA), nicotinamide (NAM), nicotinic acid riboside (NAR), and the oxidized and reduced forms of nicotinamide mononucleotide (NMN and NMNH) and nicotinamide riboside (NR and NRH).
Niacin: General term used to define vitamin B3 and derivatives, including nicotinic acid, nicotinamide, and related compounds, such as nicotinamide riboside. Vitamin B3 is found in a wide variety of foods. Many countries have food fortification programs with niacin to prevent pellagra.
Pellagra: A disease caused by deficient intake of vitamin B3 (niacin). Pellagra is also known as the disease of the four D’s, as it is clinically manifested by photosensitive dermatitis, diarrhea, dementia, and, eventually, death.
NAD(H): The oxidized (NAD^+^) and reduced (NADH) forms of nicotinamide adenine dinucleotide are essential molecules in cellular energy metabolism due to their ability to transfer electrons. NAD^+^ is also used as a substrate by several families of enzymes, the so‐called NAD^+^ consumers, which regulate major biological processes. The central role of NAD(H) in metabolism makes these molecules crucial for cellular proper function.
Preiss–Handler pathway: This pathway, described in 1958 by Jack Preiss and Philip Handler, involves the conversion of nicotinic acid into NAD^+^ in three sequential enzymatic steps carried out by the enzymes NA phosphoribosyltransferase (NAPRT), NMN adenylyltransferases (NMNATs), and NAD^+^ synthase (NADSYN).

## Introduction

Nicotinamide adenine dinucleotide exists in two forms, including an oxidized (NAD^+^) and a reduced (NADH) form, and plays a key role in intermediary metabolism, as obligatory partner in numerous oxidation/reduction reactions. Discovered over 100 years ago by Harden & Young (Harden & Young, 1906), NAD^+^ went through a period of relative anonymity until its renaissance 20 years ago, when it was reported as an essential substrate for the activity of sirtuins, a family of NAD^+^‐dependent deacetylases which play an essential role in the regulation of energy metabolism and mitochondrial function (Houtkooper *et al,* 2012). Besides sirtuins, other NAD^+^‐consuming enzymes have been identified during the last decades. This includes the poly(ADP‐ribose) polymerase (PARP) protein family (Leung, 2017) and the cyclic ADP‐ribose (cADPr) synthases, including CD38 (Aksoy *et al,* 2006) and CD157 (Ortolan *et al,* 2019). Together, these three families of enzymes regulate major biological processes in cells (Ansari & Raghava, 2010), making NAD^+^ homeostasis vital for proper cellular functioning.

The importance of NAD^+^ for human health and disease is exemplified by the existence of genetic diseases caused by defects in the biosynthesis of NAD^+^ with often devastating consequences in terms of the clinical signs and symptoms in patients. Furthermore, the cellular dependence on NAD^+^ also becomes evident if we consider the fact that NAD^+^ depletion is a common factor in numerous diseases (Okabe *et al,* 2019). Importantly, several reports have been published in which NAD^+^ repletion by means of supplementation with NAD^+^‐enhancing molecules was successful in ameliorating the outcomes of these conditions, including neurodegenerative disorders (Liu *et al,* 2013; Zhou *et al,* 2015; Schondorf *et al,* 2018), metabolic diseases (Revollo *et al,* 2007; Yoshino *et al,* 2011; Canto *et al,* 2012; Lee *et al,* 2015; Katsyuba *et al,* 2018), and age‐related complications (Mouchiroud *et al,* 2013b; Mills *et al,* 2016; Zhang *et al,* 2016).

Although most of the research on the implications of NAD^+^ in health and disease has been carried out in animal models, NAD^+^ deficiency has also been reported to cause a number of medical conditions in humans, which can be subdivided into two groups including the primary and secondary deficiencies of NAD^+^ homeostasis. The first group involves the genetically determined deficiencies of NAD^+^ synthesis, due to deleterious mutations in genes coding for enzymes directly involved in NAD^+^ biosynthesis, from both *de novo* and salvage pathways. In contrast, the second group of diseases involves alterations of NAD^+^ metabolism induced by other factors, such as increased NAD^+^ consumption and/or a dietary deficiency of NAD^+^ precursors.

In this review, we provide an overview of the current state of knowledge on human diseases caused by primary or secondary disturbances of NAD^+^ homeostasis and discuss about the therapeutic potential of NAD^+^ enhancers. However, other conditions affecting the balance between NAD^+^ and NADH, but which are not caused by a deficit in the NAD^+^ pool, i.e., the sum of NAD^+^ and NADH, are out of the scope of this review.

## Cellular NAD^+^ homeostasis

The cellular pool of NAD^+^ and NADH is tightly regulated through a careful balance between its biosynthesis—*de novo* from tryptophan or via salvage pathways from precursors—and its breakdown by NAD^+^‐consuming enzymes. The relative rates of these two processes determine the cellular availability of NAD^+^ and NADH. While the ratio between NAD^+^ and NADH plays a major role in the maintenance of redox homeostasis, in this review we will predominantly focus on NAD^+^ metabolism. For more information about the implications of the NAD^+^:NADH balance, diseases affecting this balance, and innovative therapies in this domain, we refer the readers to several reviews dealing with this topic (Ying, 2008; Teodoro *et al,* 2013; Xiao *et al,* 2018).

### NAD(H) biosynthesis and salvage

**Figure 1:**
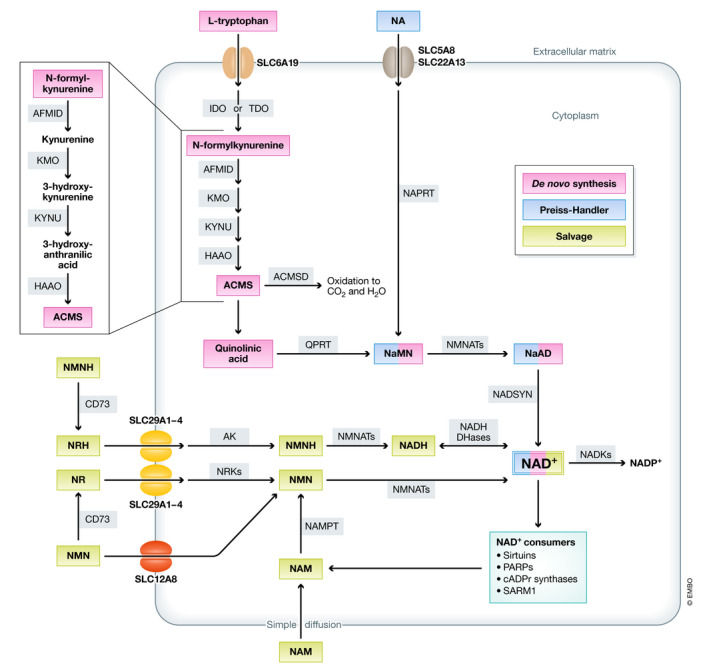
NAD^+^ synthesis pathways NAD^+^ can be synthesized *de novo* from tryptophan, through the Preiss–Handler pathway from NA or via salvage of the NAD^+^ precursors NMN, NR, NMNH, or NRH. ACMS: aminocarboxymuconate‐semialdehyde; ACMSD: aminocarboxymuconate‐semialdehyde decarboxylase; AFMID: arylformamidase; AK: adenosine kinase; CD73: cluster of differentiation 73 (5’‐nucleotidase); HAAO: 3‐hydroxyanthranilate 3,4‐dioxygenase; IDO: indoleamine 2,3‐dioxygenase; KMO: kynurenine 3‐monooxygenase; KYNU: kynureninase; NA: nicotinic acid; NADKs: NAD^+^ kinases; NaAD: nicotinic acid adenine dinucleotide; NAD^+^: nicotinamide adenine dinucleotide; NADP^+^: nicotinamide adenine dinucleotide phosphate; NADSYN: NAD^+^ synthase; NAM: nicotinamide; NaMN: nicotinic acid mononucleotide; NAPRT: nicotinate phosphoribosyltransferase; NMN: nicotinamide mononucleotide; NMNH: reduced nicotinamide mononucleotide; NMNAT: nicotinamide mononucleotide adenylyl transferase; NR: nicotinamide riboside; NRH: reduced nicotinamide riboside; NRK: nicotinamide riboside kinase; QPRT: quinolinate phosphoribosyltransferase; SLC: solute carrier transporter; and TDO: tryptophan 2,3‐dioxygenase.

NAD^+^ can be synthesized *de novo* from tryptophan or through recycling of its precursors (Fig 1). In the *de novo* pathway, dietary tryptophan is taken up into cells by neutral amino acid transporters, such as SLC6A19 (Belanger *et al,* 2018), and converted into N‐formylkynurenine by the enzyme indoleamine 2,3‐dioxygenase (IDO) or tryptophan 2,3‐dioxygenase (TDO) (Bender, 1983), after which N‐formylkynurenine is converted into α‐amino‐β‐carboxymuconate‐ε‐semialdehyde (ACMS) in a sequence of enzymatic steps carried out by arylformamidase (AFMID), kynurenine 3‐monooxygenase (KMO), kynureninase (KYNU), and 3‐hydroxyanthranilate 3,4‐dioxygenase (HAAO) (Fig 1). ACMS is then either (i) enzymatically converted into α‐amino‐β‐muconate‐ε‐semialdehyde by the enzyme ACMS decarboxylase, after which the semialdehyde is subsequently degraded to completion via multiple enzymatic steps, or (ii) spontaneously cyclized to form quinolinic acid (QA). In the latter case, QA is condensed by the enzyme quinolinate phosphoribosyl transferase (QPRT) into nicotinic acid mononucleotide (NaMN). NaMN can also be synthesized via the Preiss–Handler pathway from nicotinic acid (NA; also known as niacin or vitamin B3), which is taken up into the cell via the NA transporters SLC5A8 and SCL22A13 (Fig 1). Either way, the fate of NaMN is to be adenylated by one of the nicotinamide mononucleotide adenylyl transferases (NMNATs)—convergent enzymes to all known NAD^+^ biosynthetic pathways—to nicotinic acid adenine dinucleotide (NaAD), which is the penultimate substrate finally used by the NAD^+^ synthase (NADSYN) in combination with glutamine and ATP, to produce NAD^+^ (Houtkooper *et al,* 2010) (Fig 1).

Apart from the Preiss–Handler pathway, NAD^+^ salvage also occurs through recycling of other precursors, such as nicotinamide (NAM), which is generated by NAD^+^‐consuming enzymes, or the vitamin B3 derivative nicotinamide riboside (NR). After entering cells by simple diffusion in the case of NAM, or through the equilibrative nucleoside transporters (ENTs) SLC29A1‐4 in the case of NR, both molecules are directly converted into nicotinamide mononucleotide (NMN) by the enzyme NAM phosphoribosyltransferase (NAMPT) or the NR kinases (NRKs), respectively (Mouchiroud *et al,* 2013a). Interestingly, NMN can also act as an external NAD^+^ precursor via extracellular conversion to NR by the ectoenzyme 5’‐nucleotidase CD73 (Garavaglia *et al,* 2012; Grozio *et al,* 2013) or through specific transport via the recently described SLC12A8 transporter (Grozio *et al,* 2019).

Until very recently, six molecules were considered to act as extracellular NAD^+^ precursors including tryptophan, NA, NAM, nicotinic acid riboside (NaR), NMN, and NR (Tempel *et al,* 2007; Canto *et al,* 2015; Rajman *et al,* 2018). However, the current set of NAD^+^ precursors has recently been expanded with two new molecules, including the reduced forms of NMN (NMNH) and NR (NRH). In contrast to their oxidized counterparts, NMNH and NRH are efficiently converted to NAD^+^ via a new NAMPT and NRK‐independent pathway (Giroud‐Gerbetant *et al,* 2019; Yang *et al,* 2019; Zapata‐Pérez *et al,* 2021). In this new metabolic route, NMNH is cleaved by CD73 to NRH, which is then taken up by the same transporters (ENTs) as NR. Inside the cell, NRH is phosphorylated back to NMNH by adenosine kinase (AK), after which NMNH is finally converted into NADH by the NMNATs, which is then interconverted into NAD^+^ by the large variety of NADH dehydrogenases.

Finally, NAD^+^ can be subjected to one last phosphorylation step driven by the cytosolic NAD^+^ kinase NADK1 or the mitochondrial NADK2, both producing NADP^+^, which, together with NADPH, is essential for the maintenance of the redox balance and the biosynthesis of fatty and nucleic acids (Ying, 2008), among others (Fig 1).

Combined, the *de novo*, Preiss–Handler and NAD^+^ salvage pathways catalyze the formation of NAD(H), continuously counteracting the loss of NAD(H) by the three known families of NAD^+^‐consuming enzymes.

### NAD^+^‐consuming enzymes

Three families of proteins are classically considered as the main consumers of NAD^+^ through their enzymatic reactions, i.e., (i) sirtuins (SIRTs), (ii) poly(ADP‐ribose) polymerases (PARPs), and (iii) cyclic ADP‐ribose (cADPr) synthases, all of them generating NAM as a result of NAD^+^ utilization (Figure 1). More recently, sterile alpha and TIR motif‐containing 1 (SARM1) has been identified as an enzyme with NAD^+^‐cleavage activity in neurons, establishing a new family of NAD^+^‐consuming enzymes (Essuman *et al,* 2017). Finally, the Nudix family of hydrolases might also play a role in NAD^+^ homeostasis in mammals, as they cleave NADH to NMNH and AMP (Abdelraheim *et al,* 2003, 2017). However, there is only *in vitro* evidence of their activity, and their role in maintaining NAD^+^ homeostasis under physiological conditions remains unknown.

Sirtuins are a family of NAD^+^‐dependent protein deacetylases that, in mammals, consists of seven members (SIRT1 to SIRT7) with different subcellular locations (Michishita *et al,* 2005; Houtkooper *et al,* 2012). Sirtuins act as metabolic regulators that trigger cellular adaptations in response to changes in the cellular energy status. In line with their central role in cellular metabolism, sirtuin activation by increased NAD^+^ bioavailability has proven beneficial in a number of preclinical studies (Yoshino *et al,* 2011; Canto *et al,* 2012; Cerutti *et al,* 2014; Pirinen *et al,* 2014; Gariani *et al,* 2016; Jukarainen *et al,* 2016; Ryu *et al,* 2016) and has focused attention on this family of enzymes as potential therapeutic targets, also in humans (Jukarainen *et al,* 2016; Pirinen *et al,* 2020; Remie *et al,* 2020).

Similar to sirtuins, the DNA damage‐activated poly(ADP‐ribose) polymerases (PARPs) consume NAD^+^ during transfer of ADP‐ribosyl moieties to other proteins (Leung, 2017). However, from the 17 members that comprise the PARP superfamily, PARP1 and PARP2 account for almost all PARP activity, and therefore, these two enzymes are considered the main NAD^+^ consumers in cells (Bai & Canto, 2012).

A third cluster of NAD^+^‐consuming enzymes involves the family of cyclic ADP‐ribose (cADPr) synthases CD38 and CD157. By generating the second messenger cADPr through NAD^+^ cleavage, cADPr synthases are able to influence intracellular calcium fluxes, cell cycle activity, insulin signaling, and the immune response (Aksoy *et al,* 2006; Malavasi *et al,* 2008; Wei *et al,* 2014; Ortolan *et al,* 2019).

SARM1 has been reported as an important NAD^+^ consumer, with special relevance in the regulation of neuronal cell death. In fact, in response to neuronal damage, SARM1 is activated, catalyzing the hydrolysis of NAD^+^ to ADPr, cADPr, and NAM and promoting cytoskeletal degradation and axon destruction (Gerdts *et al,* 2013; Essuman *et al,* 2017).

For more extensive coverage of NAD^+^‐consuming enzymes, we refer the readers to several excellent reviews with more detailed information on sirtuins (Houtkooper *et al,* 2012; Chang & Guarente, 2014), PARPs (Schreiber *et al,* 2006; Canto *et al,* 2013; Leung, 2017), and cADPr synthases (Quarona *et al,* 2013).

### NAD(H) compartmentalization and maintenance of the redox balance

**Figure 2:**
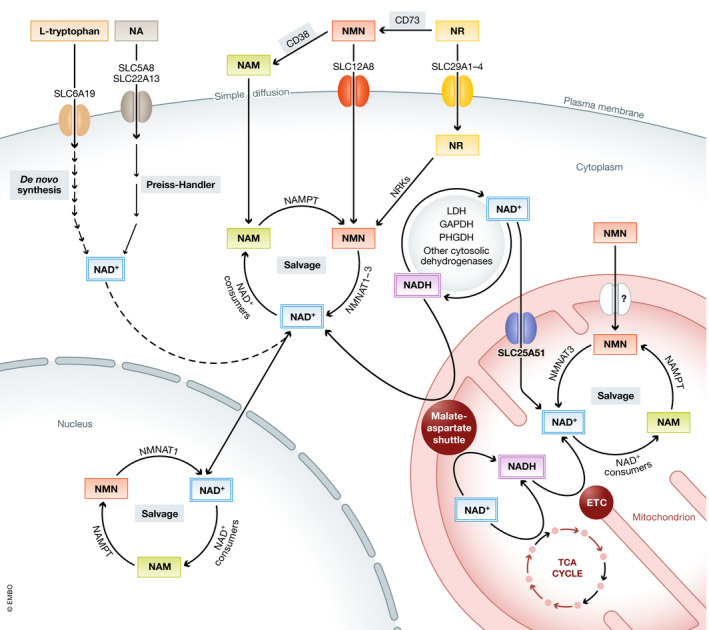
Maintenance of the interorganelle NAD(H) homeostasis Several pathways and redox systems are involved in the maintenance of cellular NAD(H) homeostasis. The NAD^+^ precursors tryptophan, nicotinic acid (NA), nicotinamide (NAM), nicotinamide mononucleotide (NMN), or nicotinamide riboside (NR) are transported into the cell via specific transporters or simple diffusion and incorporated into the cytosolic or nuclear NAD^+^ pools. Via the reactions of the cytosolic dehydrogenases or the malate‐aspartate shuttle, NAD^+^ and NADH can then be interconverted to maintain the cytosolic and nuclear NAD^+^:NADH ratios. Inside mitochondria, the malate‐aspartate shuttle works in combination with the TCA cycle and the electron transport chain (ETC) to maintain this redox balance. Besides, the mitochondrial NAD(H) pool is maintained by salvage pathways and active NAD^+^ transport through the newly discovered transporter SLC25A51. ETC: electron transport chain; GAPDH: glyceraldehyde 3‐phosphate dehydrogenase; LDH: lactate dehydrogenase; and PHGDH: phosphoglycerate dehydrogenase.

It is important to emphasize that cellular NAD^+^ homeostasis depends not only on its synthesis and utilization, but also on subcellular compartmentalization. This is not only true for the total content of NAD^+^ and NADH, but also applies to the NAD(H) redox state. Indeed, the nuclear and cytoplasmic NAD^+^:NADH ratio is 700:1, whereas that in mitochondria is much lower (8:1) (Williamson *et al,* 1967). Total NAD(H) content also differs between different cellular compartments. In fact, although NAD(H) levels are comparable between the nucleus and the cytosol (Cambronne *et al,* 2016), mitochondria can account for up to 70% of the total pool, depending on the mitochondrial volume in a particular cell type (Di Lisa & Ziegler, 2001). Although the mechanism behind this differential content of NAD^+^ plus NADH has remained unresolved, the recent identification of SLC25A51/MCART1 as an active transporter of NAD^+^ from the cytosol to mitochondria may well provide an explanation (Kory *et al,* 2020; Luongo *et al,* 2020). Furthermore, cellular NAD(H) distribution can be explained by a combination of (i) distinct subcellular expression patterns of NAD^+^ biosynthetic and consuming enzymes (Houtkooper *et al,* 2010); (ii) organelle‐specific transporters of NAD^+^ intermediates (Nikiforov *et al,* 2011; Gaudino *et al,* 2019); and (iii) a set of redox systems that interconvert NAD^+^ and NADH, such as the TCA cycle, complex I of the electron transport chain, various cytosolic dehydrogenases, or the malate‐aspartate shuttle (also known as the Borst cycle) (Canto *et al,* 2015; Yang & Sauve, 2016; Borst, 2020) (Figure 2). The presence of such a wide array of organelle‐specific mechanisms emphasizes the importance of maintaining a tight regulation of the NAD^+^ and NADH concentrations not only in the cytosol, but also at a subcellular level.

## NAD^+^ deficiency in human disease

A correct balance between NAD^+^ biosynthesis and breakdown is crucial to maintain cellular NAD^+^ homeostasis. Therefore, any imbalance between these two processes will ultimately lead to a net deficiency of intracellular NAD^+^ levels. This NAD^+^ depletion may arise from genetically determined deficiencies of NAD^+^ synthesis (primary NAD^+^ deficiencies) or from other factors that lead to a reduced NAD^+^ content (secondary NAD^+^ deficiencies). Given that putative secondary NAD^+^ deficiencies caused by deficient intake of niacin, such as pellagra, were already known to cause disease in humans long before the human genome had been sequenced and the genetic background of NAD^+^‐related disorders elucidated, we will start our discussion with these secondary deficiencies.

### Secondary NAD^+^ deficiencies

Pellagra, also known as the disease of “the four D’s”: diarrhea, dermatitis, dementia, and, eventually, death, was the first human disease associated with a deficiency in NAD^+^ precursors. Pellagra was firstly considered to be an infectious disease and reached epidemic proportions in Europe and the United States between the 18^th^ and 19^th^ centuries (Bender, 1983). It was not until 1937 when the connection between pellagra and a deficit of NA and NAM intake due to poor nutrition was made (Elvehjem *et al,* 1937). To date, this is the only known human condition caused by a deficiency of an NAD^+^ precursor in the diet.

Secondary NAD^+^ deficiencies can also have a genetic origin. A good example is glutamine synthetase deficiency due to mutations in the encoding gene (*GLUL*) (Table 1). Homozygous missense mutations in *GLUL* lead to glutamine deficiency (Haberle *et al,* 2011), which impairs the activity of the glutamine‐dependent NAD^+^ synthase (NADSYN), causing a very rare disease characterized by neonatal onset of severe encephalopathy, NAD^+^ and glutamine depletion, and an overall poor prognosis in the absence of treatment (Hu *et al,* 2015).

**Table 1:** Deleterious human variants of NAD^+^‐related genes.

Gene	Variant^1^	Clinical condition	Reference
*GLUL*	970C → A (HMZ)	Glutamine and NAD^+^ depletion, neonatal encephalopathy, hyperammonemia, poor prognosis	Hu *et al,* (2015)
*SLC6A19*	517G → A (HMZ) 718C → T (HMZ) 725T → C (HMZ) 1501G → A (HMZ) IVS8+1A (HMZ) IVS11+2G (HMZ) 908C → T; 1787_1788insG (C/HTZ)	Hartnup disorder: pellagra‐like rash, photosensitivity, ataxia, tremors.	Cheon *et al,* (2010); Kleta *et al,* (2004); Seow *et al,* (2004)
*HAAO*	483dupT (HMZ) 558G → A (HMZ)	Congenital cardiac, renal, limb, and ear‐related defects	Shi *et al,* (2017)
*KYNU*	170‐1G → T (HMZ) 468T → A; 1045_1051delTTTAAGC (C/HTZ)	Congenital cardiac, renal, limb, and ear‐related defects	Shi *et al,* (2017)
*NMNAT1*	1A → G; 769G → A (C/HTZ) 25G → A (HMZ) 37G → A; 293T → G (C/HTZ) 37G → A; 468T → C (C/HTZ) 53A → G; 769G → A (C/HTZ) 53A → G; 472G → C (C/HTZ) 59T → A; 769G→ A (C/HTZ) 98A → G (HMZ) 161C → T; 293T → G (C/HTZ) 196C → T; 709C → T (C/HTZ) 199G → T; 769G → A (C/HTZ) 205A → G; 650T → A (C/HTZ) 205A → G; 769G → A (C/HTZ) 215T → A (HMZ) 253T → C; 769G → A (C/HTZ) 271G → A (HMZ) 319G → T; 709C → T (C/HTZ) 362delA; 769G → A (C/HTZ) 362delA; 532G → A (C/HTZ) 439G → C; 769G → A (C/HTZ) 439G+1G → C; 769G → A (C/HTZ) 466G → C; 769G → A (C/HTZ) 507G → A; 710G → T (C/HTZ) 507G → A (HMZ) 518A → G; 769G → A (C/HTZ) 542A → G; 769G → A (C/HTZ) 552A → G; 769G → A (C/HTZ) 595G → T; 769G → A (C/HTZ) 617A → G; 769G → A (C/HTZ) 619C → T; 769G → A (C/HTZ) 643G → T; 769G → A (C/HTZ) 709C → T; 565delG (C/HTZ) 716T → C; 769G → A (C/HTZ) 723delA; 769G → A (C/HTZ) 731T → C; 769G → A (C/HTZ) 769G → A (HMZ) 769G → A; 817A → G (C/HTZ) 752A → G; 769G → A (C/HTZ) 838T → C (HMZ)	Early‐onset form of Leber congenital amaurosis (LCA): nystagmus, hyperopia, visual loss	Falk *et al,* (2012); Koenekoop *et al,* (2012); Nash *et al,* (2018); Perrault *et al,* (2012); Siemiatkowska *et al,* (2014)
*NADSYN1*	145T → C; 395G → T (C/HTZ) 735T → A; 1839C → G (C/HTZ) 1717G → A (HMZ) 1717G → A; 1819del (C/HTZ)	Vertebral, cardiac, renal, and limb defects	Szot *et al,* (2020)
*NAXE*	177C → A (HMZ) 196C → T; 516+1G → A (C/HTZ) 281C → A (HMZ) 653A → T; 743delC (C/HTZ) 804_807delinsA (HMZ)	Acute‐onset ataxia, delayed development, respiratory insufficiency, skin lesions, poor prognosis. Aggravated by febrile periods.	Kremer *et al,* (2016); Spiegel *et al,* (2016)
*NAXD*	44delG; 51_54delAGAA (C/HTZ) 51_54delAGAA (HMZ) 54_57delAAGA (HMZ) 101_102delTA; 318C → G (C/HTZ) 187G → A; 948_949insTT (C/HTZ) 308C → T (HMZ) 331C → T; 776T → G (C/HTZ) 839+1G → T; 922C → T (C/HTZ)	Acute‐onset ataxia, muscular hypotonia, respiratory insufficiency, skin lesion, poor prognosis. Aggravated by febrile periods.	Borna *et al,* (2020); Van Bergen *et al,* (2019); Zhou *et al,* (2020)

^1^ HMZ: homozygous and C/HTZ: compound heterozygous.

Heteroplasmic single or multiple deletions in mitochondrial DNA (mtDNA) often manifest as adult‐onset mitochondrial myopathy, a disease characterized by progressive weakness of the eye muscles, generalized muscle weakness, and fatigue (Ylikallio & Suomalainen, 2012). A very recent study has demonstrated that patients with mitochondrial myopathy also have disturbed NAD^+^ metabolism (Pirinen *et al,* 2020), with low NAD^+^ levels not only in muscle, but also in blood, pointing toward a systemic alteration of NAD^+^ metabolism. Interestingly, although no changes in the expression of PARPs, CD38, or sirtuins were detected, this study suggests that NAD^+^ depletion may be driven by impaired NAD^+^ salvage from NAM, enhanced NAM elimination, and increased activity of NAD^+^ consumers (Pirinen *et al,* 2020).

In humans, NAD^+^ deficiency can also occur during the natural process of aging (Fang *et al,* 2017). Although most of the research on the role of NAD^+^ depletion in aging has been carried out in animal models (Mouchiroud *et al,* 2013b; Yaku *et al,* 2018), mounting evidence suggests that these findings can be translated to humans. In fact, NAD^+^ levels are depleted in tissues from aged humans, such as skin (Massudi *et al,* 2012) and brain (Zhu *et al,* 2015). However, the mechanisms driving NAD^+^ depletion during human aging are not yet fully understood. In experimental model systems, it has been postulated that age‐related NAD^+^ deficiency is driven by a combination of (i) increased activity of NAD^+^‐consuming enzymes, especially PARP1 (Mandir *et al,* 1999; Liaudet, 2002; Mouchiroud *et al,* 2013b) and CD38 (Aksoy *et al,* 2006; Camacho‐Pereira *et al,* 2016), and (ii) compromised NAD^+^ biosynthesis, which is primarily caused by decreased NAMPT‐mediated NAD^+^ salvage (Revollo *et al,* 2007; Yoshino *et al,* 2011; Liu *et al,* 2012; Ghosh *et al,* 2014), but also by a decrease in the activity of the mitochondrial enzyme NMNAT3 (Son *et al,* 2016). Studies on the role of NAD^+^ biosynthetic and consuming enzymes in human aging are still in their infancy. In fact, only a few reports have shown impaired NAMPT activity or PARP1 overactivation in elderly or obese subjects (Barth *et al,* 2010; Dahl *et al,* 2010; Gaddipati *et al,* 2010; Jukarainen *et al,* 2016; Zhou *et al,* 2016), which has also been linked to thoracic aortic aneurysms (Watson *et al,* 2017). Moreover, NAMPT protein abundance decreases with age in human skeletal muscle and can be reverted by exercise training (de Guia *et al,* 2019), suggesting that physical activity may be important in the maintenance of NAD^+^ homeostasis in the aged muscle.

A role for extracellular NAMPT (eNAMPT) in maintaining NAD^+^ homeostasis during aging has also been described recently. This extracellular protein, usually considered as a proinflammatory cytokine whose increased levels in circulation have been linked to several human pathologies (Garten *et al,* 2015), can also function as a systemic NAD^+^ biosynthetic enzyme (Revollo *et al,* 2007; Yoon *et al,* 2015). A recent study shows that eNAMPT is exclusively contained in extracellular vesicles and that the levels of this extracellular protein decline with age in mice and human plasma (Yoshida *et al,* 2019). These results suggest that extracellular NAMPT plays a role in the maintenance of NAD^+^ homeostasis in human aging, especially in tissues with low levels of iNAMPT.

### Primary deficiencies of NAD^+^ synthesis

**Figure 3:**
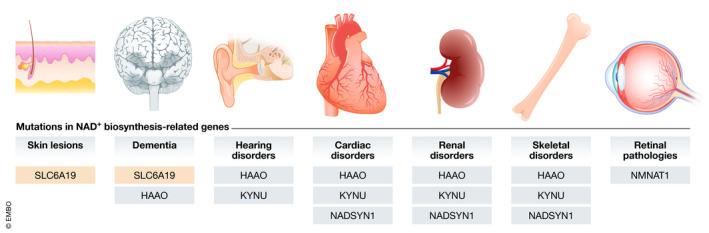
Mutations in NAD^+^ biosynthesis‐related genes cause a variety of clinical manifestations Impaired tryptophan transport through defects in the *SLC6A19* transporter is manifested as skin lesions and dementia. Dementia is also present in patients with mutations in the *HAAO* gene. Impairment of NAD^+^ synthesis, either at its upstream enzymes (HAAO and KYNU) or in the distal part of the pathway (NADSYN1), leads to cardiac, renal, hearing, or skeletal (malformations) disorders. Mutations in *NMNAT1* are the only known cause for retinal pathology associated with NAD^+^ deficiency in humans.

The primary NAD^+^ deficiencies include the inherited disorders due to mutations in genes coding for one of the enzymes involved in NAD^+^ biosynthesis, affecting either *de novo* or salvage pathways. A summary of the different diseases known late 2020 and the affected genes and tissues is provided in Table 1 and Fig 3.

Deficient intake of the NAD^+^ precursors NA and NAM is the cause of pellagra, suggesting a role of downstream NAD^+^ synthesis pathways in the onset of this disease. Pellagra can also have a genetic basis. Indeed, homozygous or compound heterozygous mutations in the neutral amino acid transporter *SLC6A19* (Table 1), which is required for tryptophan transport, cause a pellagra‐like disorder known as Hartnup disease (Kleta *et al,* 2004; Seow *et al,* 2004; Cheon *et al,* 2010), a syndrome with similar symptoms to niacin deficiency (Tahmoush *et al,* 1976). Indeed, expression of the wild‐type human *SLC6A19* in *Xenopus laevis* oocytes showed that this transporter is able to incorporate neutral amino acids in a sodium‐dependent manner. In contrast, the expression of deleterious variants of *SLC6A19* showed impaired leucine uptake, providing evidence that reduced amino acid transport through SLC6A19 is indeed the causative factor in Hartnup disease (Kleta *et al,* 2004; Seow *et al,* 2004).

Once tryptophan enters cells, it is converted into quinolinic acid (QA) in a process involving five enzymatic steps (Fig 1), two of which are carried out by the enzymes HAAO and KYNU. In a study carried out in four different families, it was reported that homozygous loss‐of‐function variants of the *HAAO* or *KYNU* genes, or compound heterozygous variants of *KYNU* (Table 1), led to reduced plasma NAD^+^ and NADH levels, and were associated with a number of clinical abnormalities, including defects in vertebral segmentation, renal hypoplasia, and other congenital malformations (Shi *et al,* 2017). The NAD^+^ decline was accompanied by large increases in the concentrations of the HAAO substrate 3‐hydroxyanthranilic acid in *HAAO*‐deficient patients, and the KYNU substrate 3‐hydroxykynurenine in *KYNU*‐deficient patients, which is in line with a block in the NAD^+^ synthesis pathway at the level of the two corresponding enzymes (Fig 1). Interestingly, follow‐up experiments in mice demonstrated that it is the loss of embryonic NAD^+^ and not a deficit in maternal NAD^+^ that leads to embryonic defects, and that niacin supplementation resolves such defects. These results provide further support suggesting that NAD^+^ homeostasis is essential for embryonic development, and emphasize the potential therapeutic benefit that niacin or other NAD^+^ precursors may have for humans suffering from these conditions.

The next step in the *de novo* synthesis of NAD^+^ is carried out by the enzyme QPRT, which converts QA, a potent excitotoxic and convulsant agent (Lugo‐Huitron *et al,* 2013), into NaMN. Although specific mutations affecting the *QPRT* gene have not yet been associated with human disease, upregulation of the kynurenine pathway and a consequent increase in QA levels have been identified in Alzheimer’s disease patients (el‐Defrawy *et al,* 1986; Guillemin *et al,* 2005). In addition, decreased QPRT activity, which also leads to QA accumulation, has been reported in brain tissue from individuals with epilepsy but not in normal human brain tissue (Feldblum *et al,* 1988), suggesting that some neurological disorders could be caused by impairment in QPRT function. However, whether QPRT impairment affects NAD^+^ levels in these patients has remained unknown.

From NaMN, the *de novo* pathway continues via adenylation to NaAD by the NMNATs, which are central enzymes also in the NAD^+^ salvage pathways (Fig 1). Among the three known NMNAT enzymes, mutations in the nuclear NMNAT isoform 1 have been identified in several studies as one of the many causes of an early‐onset and progressive form of Leber congenital amaurosis (LCA), a severe blinding retinal disease (Falk *et al,* 2012; Koenekoop *et al,* 2012; Perrault *et al,* 2012; Siemiatkowska *et al,* 2014; Nash *et al,* 2018). In these studies, individuals with LCA were found to carry homozygous or compound heterozygous nonsense, read‐through, or missense mutations predicted to cause partial loss of function of NMNAT1 (Table 1). Interestingly, these *in silico* predictions were validated through the detection of lower concentrations of NAD^+^ in red blood cells (Koenekoop *et al,* 2012) and fibroblasts (Falk *et al,* 2012) of affected individuals compared to controls, although in the latter case this difference was only borderline significant (*P* = 0.067). In these two studies, *in vitro* assays using wild‐type and mutant NMNAT1 proteins were also carried out, confirming a significantly reduced enzymatic activity of the most representative variants of NMNAT1 (Falk *et al,* 2012; Koenekoop *et al,* 2012). Interestingly, biochemical characterization of NMNAT1 mutants in a later study suggested that reduced NMNAT enzymatic activity may not be the leading cause of LCA onset since most of the LCA‐associated NMNAT1 variants did not lead to lower enzyme activities (Sasaki *et al,* 2015). At the same time, the secondary structure of many mutants was less stable, which led the authors to suggest that a combination of reduced activity and decreased protein stability under stressful conditions should be considered as the mechanism for retinal degeneration mediated by mutations in NMNAT1 (Sasaki *et al,* 2015).

The final step in the *de novo* NAD^+^ synthesis pathway is catalyzed by the glutamine‐dependent enzyme NADSYN. A study in five individuals with bi‐allelic deleterious variants in *NADSYN1* (Table 1) reported the presence of vertebral, cardiac, renal, and limb defects derived from NAD^+^ deficiency (Szot *et al,* 2020). Via a genetic complementation approach in *Saccharomyces cerevisiae* lacking the *NADSYN1* homolog *QNS1*, recombinant expression of wild‐type human *NADSYN1* was able to rescue the growth rate deficiency and to restore NAD^+^ levels in the absence of the NAD^+^ precursor NR. In contrast, the NADSYN1 p.Ala573Thr variant, which represents the most common variant identified among the patients, failed to do so under the same conditions, suggesting that the p.Ala573Thr‐variant is truly causative. These results were further corroborated by measurement of NAD^+^ synthetase activity using affinity‐purified proteins. Interestingly, *in vitro* analysis showed that mutations in both the NAD^+^‐synthase and the glutaminase domains of the protein are expected to result in a reduced capacity to generate NAD^+^ in comparison with the wild type, clearly indicating that bi‐allelic loss‐of‐function variants of *NADSYN1* are a cause of congenital NAD^+^ deficiency.

Impairment of *de novo* NAD^+^ biosynthesis at different levels is undoubtedly the cause of a variety of clinical conditions in humans. Therefore, it is tempting to speculate that loss‐of‐function mutations in other genes involved in NAD^+^ synthesis may also lead to similar conditions. These genes would include the different tryptophan transporters, such as the L‐amino acid transporters *LAT1* and *LAT2* (Kudo & Boyd, 2001; Vumma *et al,* 2011), or enzymes of the kynurenine pathway, such as *IDO*, *TDO*, *AFMID,* or *KMO*.

Importantly, loss of activity of NAD^+^ synthesizing enzymes may not be the only underlying cause of NAD^+^ deficiencies. Indeed, the high activity of α‐amino‐β‐carboxymuconate‐semialdehyde (ACMS) decarboxylase (ACMSD), an enzyme that decarboxylates ACMS, an upstream metabolite of quinolinic acid (QA), is considered a key regulator of the differences in dietary niacin requirements among species (Ikeda *et al,* 1965). Interestingly, overexpression of human ACMSD in mice generates a strong dependency on dietary niacin, which leads to NAD^+^ deficiency when these dietary requirements are not met (Palzer *et al,* 2018). Therefore, gain‐of‐function mutations in this enzyme might also contribute to reduced NAD^+^ synthesis in niacin‐deficient diets.

NAD^+^ salvage from precursors is also a main contributor to the total pool of NAD^+^ in cells (Houtkooper *et al,* 2010), although the only enzyme involved in NAD^+^ salvage that has been linked to human disease is NMNAT1, which also plays a role in the *de novo* NAD^+^ synthesis pathway. However, no specific mutations in other essential enzymes for NAD^+^ salvage have been reported to cause NAD^+^ deficiencies in humans so far. This includes (i) the NR, NMN, or NA transporters *SLC29A1‐4*, *SLC12A8*, *SLC5A8,* and *SLC22A13*, respectively, (ii) the NA, NAM, and NR‐recycling enzymes *NAPRT*, *NAMPT*, and *NRK*, (iii) the NRH‐processing enzyme *AK*, involved in NRH conversion to NAD^+^ (Giroud‐Gerbetant *et al,* 2019; Yang *et al,* 2020) (Fig 1), or (iv) the recently discovered mammalian mitochondrial NAD^+^ transporter *SLC25A51* (Kory *et al,* 2020; Luongo *et al,* 2020) (Fig 2).

### Other alterations of NAD^+^ metabolism

**Figure 4:**
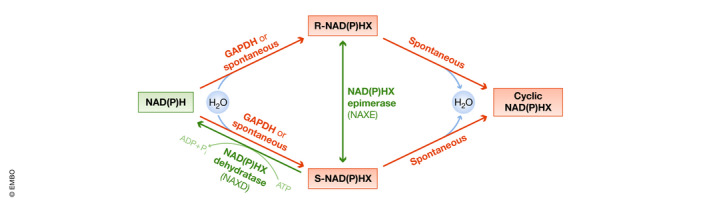
NAD(P)HX production and repair systems Via the activity of GAPDH or spontaneously under mild acidic conditions or high temperatures, NADH and NADPH can be hydrated to the R or S forms of their toxic metabolites NAD(P)HX, which can be irreversibly converted to cyclic NAD(P)HX. To avoid the accumulation of these potentially harmful intermediates, R‐NAD(P)HX are epimerized to their S forms through the activity of NAXE. S‐NAD(P)HX is then detoxified to NAD(P)H via the ATP‐dependent activity of NAXD.

Whereas research on NAD^+^ metabolism and human disease has been mainly focused on its biosynthesis and degradation, the pathological implications of the damage and repair mechanisms of NAD^+^ metabolites have only recently been considered. NADH and NADPH can be damaged by hydration of one of the double bonds of the nicotinamide ring under specific stress conditions, such as increased temperature or acidic pH, leading to the formation of the aberrant forms NADHX and NADPHX (Fig 4). NADHX can also be formed by the activity of glyceraldehyde 3‐phosphate dehydrogenase (Rafter *et al,* 1954). NAD(P)HX can spontaneously react to cyclic NAD(P)HX in an irreversible way (Fig 4). The resulting damaged/aberrant cofactors cannot act as electron carriers, and inhibit key dehydrogenase enzymes, at least *in vitro* (Yoshida & Dave, 1975; Prabhakar *et al,* 1998). To prevent their toxic effects, NADHX and NADPHX are detoxified by the sequential activity of the NAD(P)HX epimerase (NAXE), which converts the R‐NAD(P)HX epimers to their S forms, and the NAD(P)HX dehydratase (NAXD), which converts S‐NAD(P)HX back to NAD(P)H in an ATP‐dependent manner.

Inherited deficiencies of NAXE or NAXD cause fever‐induced, severe multisystemic disorders, including ataxia, cerebellar edema, respiratory insufficiency, and skin lesions, followed by rapid neurological deterioration and premature demise (Kremer *et al,* 2016; Spiegel *et al,* 2016; Van Bergen *et al,* 2019; Borna *et al,* 2020; Zhou *et al,* 2020). In two different studies with a total of eleven subjects, homozygous or compound heterozygous mutations in *NAXE* caused devastating neurological disorders (Kremer *et al,* 2016; Spiegel *et al,* 2016). While NAD(H) levels were unaltered in NAXE‐deficient fibroblasts, the R and S epimers of NADHX were found to accumulate, together with its cyclic form, for which no repair system is known (Kremer *et al,* 2016). This was accompanied by reduced activity of respiratory chain complex I and pyruvate oxidation in isolated mitochondria from fresh muscle biopsies (Kremer *et al,* 2016). Together with the accumulation of lactate measured in cerebrospinal fluid, these results clearly point toward impaired mitochondrial energy metabolism in NAXE deficiency.

Mutations in the *NAXD* gene have also been associated with accumulation of aberrant metabolites and neurological disorders. Three independent studies have demonstrated the consequences of NAXD deficiency (Van Bergen *et al,* 2019; Borna *et al,* 2020; Zhou *et al,* 2020), which resemble those found in patients with NAXE deficiency. These include accumulation of NADHX but unaltered NAD(P)H levels (Van Bergen *et al,* 2019), complex I deficiencies (Van Bergen *et al,* 2019; Borna *et al,* 2020), and reduced cytochrome *c* oxidase activity (Van Bergen *et al,* 2019). Interestingly, lentiviral transduction of the cytosolic or mitochondrial forms of NAXD completely reversed NADHX accumulation in patient fibroblasts.

*In vitro*, mutated variants of NAXD showed reduced protein expression (Borna *et al,* 2020) and loss of enzymatic activity, especially at temperatures higher than 30 °C (Van Bergen *et al,* 2019), suggesting that protein instability at relatively high temperatures may be the molecular mechanism behind fever episodes as common inductors of these diseases.

Altogether, the different studies presented in this review illustrate the importance of maintaining NAD^+^ and NADH homeostasis not only through a careful balance between their synthesis, consumption, and interconversion, but also through essential repairing mechanisms that detoxify their aberrant forms.

## Therapeutic strategies aimed at alleviating NAD^+^ deficiency in humans

The identification of NAD^+^ deficiency as the underlying cause of several human pathologies makes NAD^+^ metabolism an appealing therapeutic target. In fact, during the last decade, strategies to increase NAD^+^ levels to fight disease have attracted the attention of the research community (Houtkooper & Auwerx, 2012; Katsyuba & Auwerx, 2017; Connell *et al,* 2019).

Strategies to boost NAD^+^ levels fall into two categories: (i) inhibition of NAD^+^ consumption, and (ii) stimulation of NAD^+^ biosynthesis. Inhibition of NAD^+^ consumption has been proven effective in raising NAD^+^ levels in preclinical studies (Bai *et al,* 2011; Escande *et al,* 2013; Pirinen *et al,* 2014; Haffner *et al,* 2015). However, in humans this approach has mainly focused on the use of non‐specific CD38 inhibitors, such as quercetin, to improve cardiometabolic parameters (Pfeuffer *et al,* 2013; Dower *et al,* 2015; Menezes *et al,* 2017), or the administration of PARP inhibitors for the treatment of ovarian and breast cancers (Murthy & Muggia, 2019), but their NAD^+^‐boosting effects are still to be determined. The approach that has attracted most attention in clinical research involves stimulation of NAD^+^ synthesis by supplementation of NAD^+^ precursors. In fact, a large number of clinical trials have been carried out with the NAD^+^ precursors NA, NAM, NR, and, to a much lesser extent, NMN (Connell *et al,* 2019; Katsyuba *et al,* 2020).

The therapeutic effects of NA and NAM in humans have long been known, as they were established as the curative agents for pellagra and the pellagra‐like disorder Hartnup disease (Smith *et al,* 1937; Elvehjem *et al,* 1974; Patel & Prabhu, 2008). Apart from its role as an NAD^+^ precursor, the metabolic effects triggered by NA can also occur through its interaction with the G_i_ protein‐coupled receptor GPR109A (HM74A in humans). Through this mechanism, NA and its derivative acipimox act as lipid‐ and cholesterol‐lowering agents in humans (Sirtori *et al,* 1981; Taskinen & Nikkila, 1988; Berge & Canner, 1991; Brown *et al,* 2001; Westphal *et al,* 2006). Moreover, these molecules have been described to have potential benefits for chronic kidney disease (Restrepo Valencia & Cruz, 2008; Jin Kang *et al,* 2013) and the physiological functioning of mitochondria in muscle of subjects with type 2 diabetes (van de Weijer *et al,* 2015). NA supplementation has also been proposed to alleviate the relapse of the skin lesions in NAXD deficiency, although this study was limited to one subject and a 4‐month follow‐up period (Zhou *et al,* 2020). Interestingly, NA also restored systemic NAD^+^ deficiency, leading to improved muscle performance in patients with mitochondrial myopathy (Pirinen *et al,* 2020). In fact, the latter study is the first to demonstrate that induction of NAD^+^ levels improves muscle performance in human subjects.

The therapeutic potential of the NAD^+^ precursors NR and NMN has also been explored in a variety of clinical trials, especially in the case of NR. In contrast to NA, which induces a painful flushing reaction (Benyo *et al,* 2005), neither NMN nor NR have been reported to cause any adverse effect (Conze *et al,* 2019), even after a single oral dose of 0.5 g of NMN (Irie *et al,* 2020) or supplementation with NR for 3 months at doses as high as 2 g per day (Dollerup *et al,* 2018). However, the promising therapeutic effects observed in preclinical animal models have so far been difficult to translate to humans, where the beneficial effects of NMN and NR are mild. In fact, although NR lowers circulating inflammatory cytokines (Elhassan *et al,* 2019), increases acetylcarnitine concentrations in skeletal muscle, and increases the sleeping metabolic rate to some extent (Remie *et al,* 2020), it has no effects on overall activity, exercise performance, motor function, or mitochondrial bioenergetics (Dollerup *et al,* 2018; Martens *et al,* 2018; Elhassan *et al,* 2019; Dollerup *et al,* 2020). Furthermore, NR proved unable to improve glucose tolerance, β‐cell secretory capacity, and incretin hormone secretion in response to a glucose challenge in nondiabetic obese individuals (Dollerup *et al,* 2019). This lack of beneficial effects in humans may be in part related to the inability of NMN and NR to enhance NAD^+^ levels in human tissues. Admittedly, although an increase in whole blood NAD^+^ content was reported upon NR administration (Trammell *et al,* 2016; Airhart *et al,* 2017), supplementation with this precursor has failed to increase NAD^+^ in other tissues, such as muscle (Elhassan *et al,* 2019; Dollerup *et al,* 2020), even after administration of 1 g per day during 6 weeks (Remie *et al,* 2020). Such inefficacy of NMN and NR in enhancing NAD^+^ levels in humans could be due to the duration of the studies, which may be insufficient to achieve a clinical benefit, or with the experimental setup, which has been mainly focused on healthy subjects with normal basal NAD^+^ levels.

## Conclusions and future directions

The recognition that NAD^+^ deficiency is linked to human disease has led to a marked interest in this molecule and burgeoning of this broad and exciting field, even though the full translational and therapeutic power of repletion therapies remains to be explored. We expect that a considerable number of human diseases caused by monogenic defects in NAD^+^ metabolism remain to be discovered. Also for common diseases, it seems likely that additional pathologies in which reduced NAD^+^ plays a role in the pathophysiology will be discovered. Such findings would give way to new therapeutic strategies, such as supplementation with NAD^+^ precursors.

Future studies will need to elucidate the therapeutic potential and effects of NAD^+^ replenishment therapies for NAD^+^ deficiency conditions, especially now that NAD^+^ has arisen as the underlying cause of a number of different human diseases. Available preclinical and clinical data are promising though, as NA has already been proven to ameliorate systemic NAD^+^ deficiency in humans caused by mitochondrial myopathy (Pirinen *et al,* 2020) and to counteract the embryogenic defects of pathogenic variants of *Haao* and *Kynu* in mice (Shi *et al,* 2017). NAD^+^ enhancers are, therefore, a very interesting therapeutic option for those patients in whom impaired NAD^+^ metabolism is detected. This could apply not only to HAAO‐ and KYNU‐deficient patients, but also to other disorders such as those caused by a deficiency of GLUL (Hu *et al,* 2015) or NADSYN (Szot *et al,* 2020), which could be overcome by leveraging the NADSYN1‐independent recycling pathway from NAM, NMN, or NR.

Although attempts to replenish NAD^+^ content with NA in fibroblasts from a patient with Leber congenital amaurosis caused by *NMNAT1* mutations were unsuccessful (Falk *et al,* 2012), the potential of other NAD^+^‐boosting molecules warrants further research. Indeed, patients suffering from this disorder could benefit from this therapy, as supplementation with NAD^+^ precursors or NAD^+^‐consumption inhibitors could ensure enough substrate for NMNAT1 to overcome its reduced enzymatic function.

In light of the current evidence on the use of NAD^+^ replenishment therapies to treat human disease, future clinical trials should aim at establishing which precursor is best for each of these conditions. Surely, there is still a long way to go until a definitive cure for NAD^+^ deficiencies is found, but all the odds are on our side.

## Pending issues

- Identifying underlying NAD^+^ deficiencies in common and/or NAD^+^ metabolism‐related pathologies could lead to new therapeutic strategies for these diseases.
- NAD^+^‐replenishment therapies should be tested in patients with suspected or confirmed NAD^+^ deficiency, which includes HAAO‐ and KYNU‐deficient patients
- The pathophysiological role of many genes involved in NAD^+^ metabolism has not yet been elucidated. New studies attempting to associate mutations in these genes to human disease would be specially interesting.
- New therapeutic avenues aimed to alleviate the consequences of NAD(P)HX accumulation should be explored, e.g., through gene replacement therapies.

## Author contributions

RZ‐P, RJAW, CDMvK, and RHH contributed to the conception, the writing, and the editing of the manuscript.

## Conflict of interest

The authors declare that they have no conflict of interest.
